# Melphalan flufenamide inhibits osteoclastogenesis by suppressing proliferation of monocytes

**DOI:** 10.1016/j.bonr.2021.101098

**Published:** 2021-06-07

**Authors:** Konstantin Byrgazov, Thomas Lind, Annica J. Rasmusson, Claes Andersson, Ana Slipicevic, Fredrik Lehmann, Joachim Gullbo, Håkan Melhus, Rolf Larsson, Mårten Fryknäs

**Affiliations:** a Oncopeptides AB, Stockholm, Sweden; b Department of Medical Sciences, Uppsala University, SE-751 85 Uppsala, Sweden

**Keywords:** Osteoclastogenesis, Bone resorption, Melflufen, Melphalan

## Abstract

Myeloma bone disease is a major complication in multiple myeloma affecting quality of life and survival. It is characterized by increased activity of osteoclasts, bone resorbing cells. Myeloma microenvironment promotes excessive osteoclastogenesis, a process of production of osteoclasts from their precursors, monocytes. The effects of two anti-myeloma drugs, melphalan flufenamide (melflufen) and melphalan, on the activity and proliferation of osteoclasts and their progenitors, monocytes, were assessed in this study. In line with previous research, differentiation of monocytes was associated with increased expression of genes encoding DNA damage repair proteins. Hence monocytes were more sensitive to DNA damage-causing alkylating agents than their differentiated progeny, osteoclasts. In addition, differentiated progeny of monocytes showed increased gene expression of immune checkpoint ligands which may potentially create an immunosuppressive microenvironment. Melflufen was ten-fold more active than melphalan in inhibiting proliferation of osteoclast progenitors. Furthermore, melflufen was also superior to melphalan in inhibition of osteoclastogenesis and bone resorption. These results demonstrate that melflufen may exert beneficial effects in patients with multiple myeloma such as reducing bone resorption and immunosuppressive milieu by inhibiting osteoclastogenesis.

## 1 Introduction

Multiple myeloma (MM) is a hematological cancer characterized by malignant proliferation of monoclonal plasma cells in bone marrow. The majority of MM patients already display bone lesions at diagnosis (Terpos et al., 2018). During the disease course most of them will develop pathological fractures causing debilitating pain (Terpos et al., 2010). Excessive bone destruction is also associated with hypercalcemia, a major metabolic complication in MM patients (Oyajobi, 2007), increased morbidity and mortality in patients with MM (Terpos et al., 2013). On a cellular level, bone disease in myeloma is characterized by increased bone resorption activity mediated by osteoclasts. These are myeloid lineage cells derived from CD14-positive monocytes in a differentiation process called osteoclastogenesis. Bone lesions in MM patients are purely lytic due to local excessive osteoclastogenesis promoted by MM cells (Marino and Roodman, 2018). MM patients with bone disease show an accumulation of bone-resorbing osteoclasts in close proximity to MM cells as a result of local production of factors stimulating osteoclastogenesis (Marino and Roodman, 2018). Besides their role in bone disease, osteoclasts are also known to support myeloma cells (Yaccoby et al., 2002; Yaccoby et al., 2008; Yaccoby, 2010; Hecht et al., 2008). In addition to their support role of myeloma cell survival, osteoclasts also promote an immunosuppressive microenvironment (An et al., 2016). Several immune checkpoint molecules, and negative regulators of T cell metabolism, have previously been shown to be up-regulated during osteoclastogenesis (An et al., 2016; Tai et al., 2018). Thus, besides promoting bone disease and making a severe negative impact on the quality of life of MM patients, osteoclasts contribute to myeloma progression by supporting survival of myeloma cells and suppressing immune responses in a MM microenvironment.

Melphalan flufenamide (melflufen) is a first-in-class peptide-drug conjugate that rapidly delivers alkylating payloads to malignant cells through aminopeptidase-driven accumulation of metabolites with alkylating potential (Wickstrom et al., 2017). Currently, administration of melflufen in combination with dexamethasone is showing promising results in heavily pretreated relapsed refractory MM patients (Oriol et al., 2020; Richardson et al., 2020a; Mateos et al., 2020). Adverse effects upon melflufen administration are predominantly hematological within myeloid milieu, with thrombocytopenia and neutropenia being the most frequent high-grade complications associated with melflufen administration in MM patients (Richardson et al., 2020a). Previously, our group has shown that melflufen suppresses proliferation of myelomonocytic leukemic cells, the malignant counterpart of monocytes, which are the precursors of osteoclasts (Strese et al., 2017). Also non-leukemic monocytes were shown to be more sensitive to DNA damage compared to their precursors and differentiated progeny, macrophages (Bauer et al., 2011; Berte et al., 2020) and dendritic cells (Briegert and Kaina, 2007). Therefore, the direct effects of melflufen on human osteoclastogenesis, a process underlying myeloma bone disease, using cultured peripheral blood CD14-positive monocytes, was evaluated in this study.

## 2 Materials and methods

### 2.1 Ethical aspects

All procedures performed in studies involving human specimen were in accordance with the ethical standards of the institutional and/or national research committee and with the 1964 Helsinki Declaration and its later amendments or comparable ethical standards. Sampling and data collection was performed following informed consent, and the study was approved by the Regional Ethical Committee in Uppsala (Dnr 2007/237).

### 2.2 Materials

Recombinant human macrophage colony-stimulating factor (M-CSF, also termed CSF1) was purchased from Sigma-Aldrich (Sweden). Recombinant human soluble receptor activator of nuclear factor kappa-Β ligand (RANKL) was obtained from Peprotech (USA). Melphalan were purchased from Sigma-Aldrich (Sweden). Melflufen was received from Oncopeptides (Sweden).

### 2.3 Osteoclast progenitors

Isolation of CD14^+^ monocytes (osteoclast progenitors) was performed as previously described (Hu et al., 2010). In brief, buffy coat blood was obtained from anonymous healthy donors at Uppsala University Hospital. The peripheral blood mononuclear cells (PBMCs) were isolated by Histopaque-1077 (Sigma-Aldrich, Sweden) density gradient centrifugation according to the manufacturer's instruction. The PBMCs were re-suspended in cold PBS containing 0.5% BSA and 2 mM EDTA. The human CD14^+^ monocytes were isolated using Classical monocyte isolation kit human from Miltenyi Biotec (Germany). Human CD14^+^/CD16^−^ cells were seeded in supplemented RPMI-1640 medium with 25 ng/ml M-CSF for 2 days. By the second day, the cells were detached by incubation with Accutase at 37 °C for 10 min and removed by a scraper. Cells were cultured at 37 °C in a humidified atmosphere of 5% CO_2_ monitored in a IncucyteS3 every 4th hour. Media was changed every second day of culture, except over weekends. Before each experiment cells were counted, and viability determined by using a Cell Counter NC-250.

### 2.4 Analysis of tartrate-resistant acid phosphatase activity

Osteoclast progenitors were seeded in a 96-well plate at a density of 50,000 cells/well with supplemented RPMI-1640 medium complemented with M-CSF and/or RANKL each at 25 ng/ml. Osteoclasts were identified by measuring increasing tartrate-resistant acid phosphatase (TRAP) activity in the culture medium with an assay developed previously (Kirstein et al., 2006; Lang et al., 2001). Briefly, medium was added to the ELISA plates containing 0.1 M acetate (pH 5.2), 0.15 M potassium chloride, 0.1% triton X-100, 1 mM sodium ascorbate, 0.1 mM ammonium ferrious sulfate hexahydrate, 10 mM phosphatase substrate *p*-nitrophenyl phosphate (PNPP) and 10 mM sodium tartrate acid buffer. The plate was incubated at 37 °C for 30 min. The reaction was stopped with the addition of 0.3 M NaOH and absorbance was measured at 405 nm. TRAP catalyzes the conversion of PNPP to *p*-nitrophenol (PNP), giving a maximal absorbance at 405 nm, which corresponds to the TRAP activity in the sample. Also, TRAP+ cells containing 3 or more nuclei were considered as osteoclasts. The TRAP staining was carried out using the Acid Phosphatase, Leukocyte (TRAP) Kit (Sigma-Aldrich, Sweden).

### 2.5 Differentiation of osteoclast progenitors

Osteoclast progenitors, obtained as described above, were re-suspended to a final concentration of 0.5 million cells/ml in supplemented RPMI-1640. 100 μl of cell suspension per well (50,000 cells/well) was plated in a 96 well plate. 100 μl of drug-solution (melphalan or melflufen) in medium complemented with RANKL and M-CSF (final concentration of 25 ng/ml each) was added at 72 h upon start of the experiment. Cells were cultured at 37 °C in a humidified atmosphere of 5% CO_2_ monitored in Incucyte S3 every 4th hour. After 10 days of culture, the osteoclasts could be identified as large multinucleate cells when visualized by phase microscopy. Media was changed every second day of culture, except over weekends.

### 2.6 The Osteo Assay resorption determination

Osteoclast progenitors were seeded at 50000 cells/well in a 96 well Corning Osteo Assay Surface plate (Corning), with the control medium supplemented with M-CSF and RANKL each at 25 ng/ml, according to the manufacturer's instructions. For the resorption experiment melphalan (1–50 μM) and melflufen (0.1–10 μM) were added on day 0 and incubated for 7 days. After 7 days of culture, the osteoclasts could be identified as large multinucleate cells when visualized by phase microscopy. To analyze the surface for pit formation the medium was aspirated from the wells on day 10 and 100 μl of 14% hypochlorite solution was added. Cells were incubated with the hypochlorite solution for 5 min at room temperature. The wells were washed twice with MQ water and allowed to dry at room temperature overnight. Individual pits or multiple pit clusters were observed using a microscope at 40 to 100× magnification.

### 2.7 The bovine bone resorption assay

For measurement of bone collagen degradation osteoclast progenitors were seeded at 150000 cells/well in a 96 well containing bovine bone slices (Immunodiagnostics Systems Nordic, Denmark), with the control medium supplemented with M-CSF and RANKL each at 25 ng/ml. For the bone resorption experiment melphalan (1–50 μM) and melflufen (0.1–10 μM) were added on day 0 and incubated for 12 days. Cell media was collected and used to determine the release of C-terminal telopeptide of type I collagen (CTx) from mineralized bone slices using CrossLaps for Culture kit (Nordic Bioscience Diagnostics, Denmark) according to the manufacturer's instructions.

### 2.8 Measurement of cell viability and proliferation

The Fluorometric Microculture Cytotoxicity Assay, FMCA, described in detail previously (Lindhagen et al., 2008), was used for measurement of cell viability and proliferation. The FMCA is based on measurement of fluorescence generated from hydrolysis of fluorescein diacetate (FDA) to fluorescein by cells with intact plasma membranes. Cells were seeded in 384-well plates using the pipetting robot Biomek 4000 (Beckman Coulter Inc., Brea, CA) and cultured overnight before drugs were added by the Echo Liquid Handler 550 system (Labcyte, Sunnyvale, CA). In each plate, four columns without drugs added served as controls and one column with medium only served as blank. Cell survival, expressed as survival index (SI) is defined as fluorescence in test wells divided by fluorescence of control wells, with blank values subtracted, multiplied by 100.

### 2.9 RNA isolation and gene expression

RNA from cell cultures was isolated using RNeasy Mini Kit from Qiagen and immediately stored at −70 °C until further use. RNA purity and quality were assessed using an ND 1000 spectrophotometer (NanoDrop Tecnhologies, Wilmington, DE) and Bioanalyzer 2100 (Agilent Technologies Inc., Palo Alto, CA, USA), respectively. Clariom S gene expression Assay (ThermoFisher) was used, according to manufactures instructions, to generate gene expression data. Microarray data was processed using Transcriptome Analysis Console 4.0.2.15 (Affymetrix, Santa Clara, CA, USA). Microarray data (CEL files) was normalized using SST-RMA to compute differential expression and visualized in a principal components analysis plot with default settings. The 200 most overexpressed genes in CD14^+^ RANKL+M-CSF vs. CD14^+^- monocytes were analyzed using WikiPathways via Enrichr analysis tool (Chen et al., 2013).

### 2.10 Statistical analysis

All statistical analyses were performed using GraphPad prism. Feasibility of applying paired *t-*test was justified by testing the normality of the data against the null hypothesis that it is normally distributed.

## 3 Results

### 3.1 Culture system for osteoclastogenesis

**Fig. 1:**
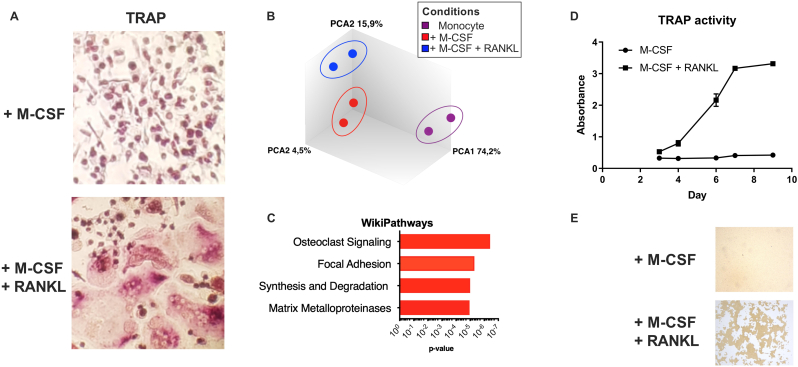
Validation of the culture system for osteoclastogenesis gene expression and resorption activity. (A) TRAP staining of CD14+-monocytes exposed to M-CSF or RANKL and M-CSF for 12 days. (B) Global gene expression analysis of unstimulated CD14+-monocytes and CD14+-monocytes exposed to RANKL and/or M-CSF for 12 days (biological replicates) visualized using principal component analysis. (C) The 200 most overexpressed genes in CD14+ RANKL+M-CSF vs. CD14+-monocytes were analyzed using WikiPathways via Enrichr pathway analysis tool, and four most highly enriched pathways are shown. (D) TRAP activity in the media is shown to follow RANKL-induced osteoclastogenesis due to specific secretion of TRAP by osteoclasts. (E) Bone degradation activity as determined by resorption in osteoplates covered with inorganic matrix phosphate. Yellowish area represents phosphate-covered scaffold whereas whiter area indicates the loss of the inorganic phosphate cover.

To test the effect of melflufen on human osteoclastogenesis and mature osteoclasts we used a validated culture system. CD14^+^ monocytes, isolated from peripheral blood, were cultured with M-CSF and RANKL for 10–14 days (Sorensen et al., 2007). Simultaneous addition of M-CSF and RANKL produced cells with a multinuclear phenotype, a typical characteristic of osteoclasts, as revealed by staining of tartrate resistant acidic phosphatase (TRAP) (Fig. 1A). Global gene expression analysis showed that addition of both M-CSF and RANKL to CD14^+^ cells produced cell populations distinct from untreated monocytes, and monotreatment with M-CSF, in reproducible manner (Fig. 1B). Moreover, cultivation of CD14^+^ monocytes with M-CSF and RANKL produced cells with gene expression indicating formation of osteoclasts according to the analysis of WikiPathways via Enricher analysis tool (Fig. 1C). Noteworthy, although TRAP activity staining reveals differentiated cells in both M-CSF and M-CSF + RANKL populations, TRAP activity in the supernatant related to osteoclast-specific secretion of the bone resorbing enzyme was only detectable upon addition of both RANKL and M-CSF, but not M-CSF alone (Fig. 1D–E). As previously shown, TRAP activity in the supernatant of osteoclast culture is associated with bone resorbing activity and not osteoclastic cell death (Kirstein et al., 2006).

**Fig. 2:**
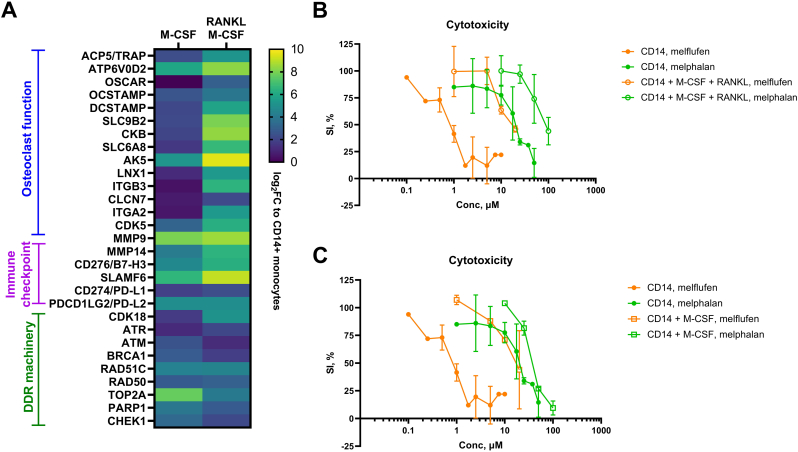
(A) Differential gene expression of several genes implicated in osteoclast function, immune suppression, and DNA damage repair. Log2-transformed fold change to undifferentiated CD14+ monocytes is shown by color-coded bars. (B–C) Drug sensitivity analysis of monocytes before and after differentiation with a combination of M-CSF and RANKL (B) or M-CSF alone (C). Inhibition of cell proliferation in the presence of melflufen is shown by orange lines, melphalan – by green lines. Undifferentiated monocytes are marked by filled circles, M-CSF + RANKL cell fraction is indicated by empty circles, M-CSF fraction – by empty squares. The results of two independent experiments are shown. SI% = survival index.

In the gene expression analysis, we observed up-regulation of several genes (*ACP5* (TRAP), *OSCAR*, *ATP6V0D2*, *DCSTAMP*, *OCSTAMP*, *ITGB3*, *CLCN7*, *AK5*, *LNX1*, *SLC9B2*) implicated in osteoclast function (Sorensen et al., 2007; Aliprantis et al., 2008; Georgess et al., 2014; Battaglino et al., 2008) (Fig. 2A). Genes encoding creatine kinase *CKB* and creatine transporter *SLC6A8* were also up-regulated in osteoclast-specific manner confirming the role of creatine kinase in osteoclast bone resorbing activity (Chang et al., 2008) (Fig. 2A). Furthermore, osteoclast-specific up-regulation of exocytosis master regulator cyclin-dependent kinase *CDK5 (*Rosales and Lee, 2006*)* was observed in this study for the first time. Moreover, we observed the up-regulation of expression of several genes encoding molecules that negatively regulate the immune response. *MMP14*, *MMP9*, *CD274* (PD-L1), *PDCD1LG2* (PD-L2), *CD276* (B7-H3), and *SLAMF6* mRNAs showed significant up-regulation upon differentiation of monocytes induced by M-CSF alone or a combination of M-CSF and RANKL (Fig. 2A).

### 3.2 Melflufen is more efficient than melphalan in negatively affecting CD14^+^ osteoclast progenitors

In line with previously published research showing that differentiation of monocytes is associated with an increase in DNA damage repair (DDR) (Bauer et al., 2011; Bauer et al., 2012), we observed up-regulation of several DDR genes (Fig. 2A). Axis CDK18/ATR/CHEK1 was previously implicated in genome stability (Barone et al., 2016), whereas TOP2A, RAD50, RAD51C were implicated in repair of DNA damage induced by alkylating agents (Eder Jr et al., 1995; Mohan et al., 2019). Indeed, cytotoxicity assays performed in this study showed that M-CSF + RANKL- and M-CSF-mediated differentiation of monocytes is associated with decreased sensitivity to melphalan flufenamide (melflufen) and melphalan (Fig. 2B–C). The half maximal inhibitory concentration (IC_50_) of melflufen increased from 1.0 μM in CD14 monocytes up to 15 μM in M-CSF-derived macrophages and M-CSF + RANKL-derived osteoclasts, while melphalan IC_50_ increased from 20 μM in monocytes up to 50 μM in macrophages and 100 μM in osteoclasts (Fig. 2).

### 3.3 Melflufen is superior to melphalan in inhibiting osteoclast function

**Fig. 3:**
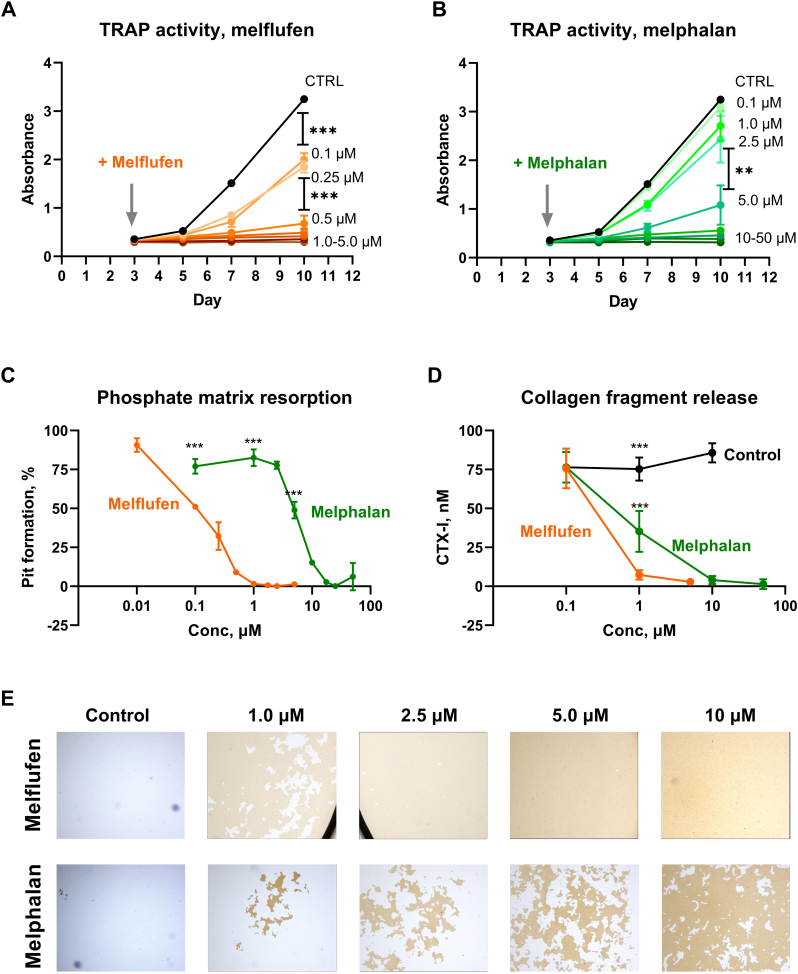
Effect of melflufen (A) and melphalan (B) on osteoclastogenesis in the presence of inorganic phosphate matrix as determined by TRAP activity secreted into the supernatant. Addition of the drugs at the day 3 of osteoclastogenesis is indicated by a vertical arrow. Drug concentrations are indicated next to the TRAP activity curves measured by absorbance as described in Materials and Methods. (C) Quantification of pit formation on the inorganic phosphate matrix upon aforementioned osteoclastogenesis on the inorganic phosphate matrix. (D) Quantification of collagen fragment release from bone slices as a result of osteoclastogeneiss in the presence of melflufen and melphalan. (E) Visualization of pit formation on the osteo plate covered by inorganic phosphate during osteoclastogenesis in the presence of indicated concentration of melflufen and melphalan. Yellowish area represents phosphate-covered scaffold whereas whiter area lost the inorganic phosphate cover. All results are presented as mean ± SD. Statistical analysis is done by paired *t-*test after testing for normality; ** - p < 0.01, *** - p < 0.001.

Next, the effect of melphalan and melflufen on the function of osteoclastogenesis was assessed. First, osteoclastogenesis in the presence of both drugs was assessed in the presence of inorganic phosphate matrix by means of TRAP activity secreted into supernatant by newly formed osteoclasts. Melflufen was able to significantly decrease TRAP secretion and osteoclast formation already at the concentrations of 0.1–0.25 μM (Fig. 3A). Addition of 0.5 μM corresponding to the achievable physiological concentration of melflufen in patients (Wickstrom et al., 2017) completely stopped osteoclastogenesis (Fig. 3A). As for melphalan, ten-fold higher concentration of 5.0 μM was required to achieve the same effect (Fig. 3B). After the assay had been stopped, the cells were washed away from the inorganic phosphate matrix and resorption of the matrix was quantified by image analysis. In concordance with the reduction of TRAP activity, addition 0.1 μM of melflufen resulted in 50% reduction of phosphate matrix resorption, while fifty-fold higher amount of melphalan (5.0 μM) was necessary to achieve the same effect (Fig. 3C, E). Another important functionality of osteoclasts, destruction of collagen matrix, was also assessed upon addition of melflufen and melphalan early in the osteoclastogenesis followed by measurement of collagen fragments (CTx) in the supernatant. Similar to previous assays, melflufen was 10-fold more active than melphalan in stopping bone resorption indicated by collagen fragment release by osteoclasts from the bone slices (Fig. 3D).

## 4 Discussion

Multiple myeloma is often accompanied by bone pain and pathological fractures affecting the quality of life and survival of MM patients. Bone pain and pathological fractures are associated with lytic bone lesions, often already detectable in MM patients at diagnosis. These bone lesions are the result of excessive osteoclastogenesis triggered by malignant plasma cells (Marino and Roodman, 2018). Increased number of osteoclasts support myeloma cell survival by secreting pro-survival factors (Yaccoby et al., 2002; Yaccoby et al., 2008; Yaccoby, 2010), direct contact (Hecht et al., 2008; Abe et al., 2004), and creating an immunosuppressive microenvironment (An et al., 2016; Tai et al., 2018). This and previous studies have shown that several immune suppressive molecules negatively affecting the immune response against the tumor are up-regulated during differentiation of monocytes and osteoclastogenesis (Hecht et al., 2008; An et al., 2016). Firstly, matrix metalloprotease 9 (MMP9), also known as 92 kDa collagenase type IV, is able to simultaneously promote both bone resorption and an immunosuppressive microenvironement (Hecht et al., 2008; Juric et al., 2018; Lavini-Ramos et al., 2017). In addition, matrix metalloprotease 14 (MMP14) promotes shedding of MHC class I Chain related molecule A (MICA), thus impairing NK and T cell anti-tumor responses (Liu et al., 2010). Other immunosuppressive molecules strongly up-regulated during monocytic differentiation and osteoclastogenesis include genes encoding B7 family members B7-H1/PD-L1, B7-H2/PD-L2, and B7-H3/CD276. The first two members of the B7 family, PD-L1 and PD-L2, are well known immune checkpoint molecules which inhibit T cell proliferation upon interaction with the PD-1 receptor on the surface of T cells, thus allowing immune escape of the tumor cells (Boussiotis, 2016). However, despite its success in solid tumors and lymphoma, clinical application of PD-1 blockade did not significantly improve survival of patients with MM (Mateos et al., 2019a; Mateos et al., 2019b), pointing at other mechanisms of immunosuppression in the MM microenvironment. B7 family member B7-H3 encoded by *CD276* gene is up-regulated during osteoclastogenesis. This immune checkpoint molecule has been shown to control CD8^+^ T cells and NK cells (Lee et al., 2017). In addition, SLAMF6 is another negative regulator of CD8^+^ T cell response (Yigit et al., 2019a; Yigit et al., 2019b) up-regulated during osteoclastogenesis. Thus, osteoclasts not only support myeloma cell growth, but they also impede immune responses through several highly expressed negative regulators of immune response including, but not limited to, MMP9, MMP14, B7-H3, PD-L1, and PD-L2. This mutually beneficial interaction between myeloma cells and osteoclasts creates a vicious cycle resulting in disease progression, and increased morbidity and mortality of patients with MM. Breaking this cycle represents an attractive clinical strategy in multiple myeloma.

Melflufen is a first-in-class peptide-drug conjugate showing promising results in relapsed/refractory MM patients (Oriol et al., 2020; Richardson et al., 2020a; Mateos et al., 2020; Richardson et al., 2020b). Addition of melflufen at early stages of osteoclastogenesis significantly impeded formation of osteoclasts, resulting in decreased bone resorption activity in vitro. The results of this study demonstrate that melflufen efficiently inhibits osteoclastogenesis, and associated bone destruction, by negatively affecting proliferation of monocytes, the precursors to osteoclasts. Noteworthy, melflufen is at least 10-fold more effective in stopping osteoclastogenesis and associated bone destruction than melphalan. Although mature osteoclasts could be significantly affected by melflufen only at very high concentrations, they have relatively short life span (Manolagas, 2000). Thus, by negatively affecting osteoclast precursors, melflufen could have a long-term effect through preventing accumulation of osteoclasts, and stopping excessive osteoclastogenesis and bone resorption, which may potentially result in decreased bone pain in MM patients. Moreover, melflufen may improve immune response against myeloma cells by removing monocytes, the precursor cells to several immunosuppressive cell populations such as osteoclasts and monocytic myeloid-derived suppressor cells (Malek et al., 2016). Synergism of melflufen with immunotherapy, an anti-CD38 monoclonal antibody daratumumab, in patients with relapsed/refractory MM, is currently under investigation in clinical trials (Oriol et al., 2020; Mateos et al., 2020).

## CRediT authorship contribution statement

KB and MF wrote the main manuscript. KB, CA, MF, AS, AR, FL, TL, HM, JG, RL corrected the manuscript. AR, TL, HM performed osteoclast-related experiments including culture of osteoclasts and their precursors, osteoclast differentiation, bone resorption and collagen release assays. MF, CA, KB performed RNA isolation and gene expression analysis. KB prepared all the figures and files for the submission.

## Declaration of competing interest

KB, AS, FL are employed by Oncopeptides AB. FL, JG, RL have an equity in Oncopeptides AB. JG and RL are founders of Oncopeptides AB. JG is a consultant for Oncopeptides AB. JG is founder of Theradex Oncology. All other authors declare no conflict of interests.
